# A Spike-Based Neuromorphic Architecture of Stereo Vision

**DOI:** 10.3389/fnbot.2020.568283

**Published:** 2020-11-13

**Authors:** Nicoletta Risi, Alessandro Aimar, Elisa Donati, Sergio Solinas, Giacomo Indiveri

**Affiliations:** Institute of Neuroinformatics, University of Zurich, Eidgenössische Technische Hochschule Zurich, Zurich, Switzerland

**Keywords:** neuromorphic, event-based processing, event-based sensing, stereo vision, asynchronous computation

## Abstract

The problem of finding stereo correspondences in binocular vision is solved effortlessly in nature and yet it is still a critical bottleneck for artificial machine vision systems. As temporal information is a crucial feature in this process, the advent of event-based vision sensors and dedicated event-based processors promises to offer an effective approach to solving the stereo matching problem. Indeed, event-based neuromorphic hardware provides an optimal substrate for fast, asynchronous computation, that can make explicit use of precise temporal coincidences. However, although several biologically-inspired solutions have already been proposed, the performance benefits of combining event-based sensing with asynchronous and parallel computation are yet to be explored. Here we present a hardware spike-based stereo-vision system that leverages the advantages of brain-inspired neuromorphic computing by interfacing two event-based vision sensors to an event-based mixed-signal analog/digital neuromorphic processor. We describe a prototype interface designed to enable the emulation of a stereo-vision system on neuromorphic hardware and we quantify the stereo matching performance with two datasets. Our results provide a path toward the realization of low-latency, end-to-end event-based, neuromorphic architectures for stereo vision.

## 1. Introduction

Biological and artificial binocular visual systems rely on stereo-vision processes to merge the visual information streams. This implies solving the stereo-matching problem, i.e., finding correspondent points in two slightly shifted views (Cumming and Parker, 1997). Typical applications in robotics that can benefit from stereo vision include navigation in unknown environments, object manipulation, and grasping. However, current machine-vision approaches still lag behind their biological counterpart mainly in terms of bandwidth and power consumption (Tippetts et al., 2016; Steffen et al., 2019). Classical methods are based on frame-based vision sensors. The main challenges of frame-based algorithms are spatial redundancy and temporal information loss due to the intrinsic nature of fixed-rate processing. This affects latency, throughput, and power consumption, making frame-based approaches difficult to integrate into mobile platforms.

Biological systems, on the other hand, seem to efficiently solve the stereo-matching problem by using space-variant and asynchronous space-time sampling (Steffen et al., 2019). Space-variant resolution refers to a non-uniform distribution of retinal photoreceptors, with higher density in the center (fovea) and a decreasing density toward the periphery. Asynchronous instead refers to event-driven, self-timed sensing and processing. Therefore, a massively parallel, asynchronous, event-based chain, from sensing to processing, seems to be a promising solution for more robust and efficient architectures of stereo vision.

In this context, neuromorphic hardware has proven to be an effective substrate (Chicca et al., 2014; Indiveri et al., 2015). To date, the emerging field of event-based stereo vision has shown successful approaches that interface Spiking Neural Networks (SNNs) with neuromorphic event-based sensors, also referred to as “event cameras,” in order to build real-time event-based visual processing systems (Mahowald, 1994a; Osswald et al., 2017). Inspired by the retinal ganglion cells, the neuromorphic vision sensors broadcast information, independently for all the pixels, only in response to significant changes in illumination, which results in a low-power, low-latency, event-driven, and sparse input stream (Lichtsteiner et al., 2008; Posch et al., 2010; Berner et al., 2013). Spiking neurons, in turn, can process signals using temporal information, and therefore, can take full advantage of an event-based input stream to solve the stereo-matching problem. However, although several biologically-inspired implementations of stereo vision (Mahowald, 1994b; Piatkowska et al., 2013, 2017; Dikov et al., 2017; Osswald et al., 2017; Kaiser et al., 2018) have extensively been explored, only a few solutions fully exploit the advantages of parallel computation, with an end-to-end neuromorphic architecture that can replace traditional Von Neumann architectures. In Dikov et al. (2017), the first scalable architecture of the Marr and Poggio cooperative network (Marr and Poggio, 1976, 1977, 1979) is implemented on the SpiNNaker platform (Furber et al., 2014). Despite the short latency (2 ms) of the network and the portable design, the reported power consumption of the neuromorphic implementation (90 W for a 3-board SpiNNaker machine) makes it difficult to integrate in mobile or autonomous applications. More recently, Andreopoulos et al. (2018) proposed the first fully end-to-end stereo pipeline, implemented on multiple TrueNorth processors (Sawada et al., 2016). The architecture achieves a 200 × improvement, compared to Dikov et al. (2017), in terms of power per pixel per disparity map (0.058 mW/Pixel). Both solutions, however, emulate the cooperative stereo network on digital hardware. Inspired by biological neurons, analog neuromorphic circuits, by contrast, can potentially lead to more promising solutions for low-power, yet noisy, computation.

Following up on the work from Osswald et al. (2017), we present an end-to-end neuromorphic architecture of cooperative stereo vision implemented on mixed analog/digital neuromorphic hardware. Compared to the previous work, here we replaced the mixed-signal Very Large Scale Integration (VLSI) ROLLS chip (Qiao et al., 2015) with a scalable multi-core design (Moradi et al., 2018). Moreover, the proposed solution shifts the event-based computation directly on chip and provides a more robust, biologically-inspired coincidence detection mechanism. In the next section, we describe the digital interface between the sensing and the processing stage. Then, we present the neuromorphic implementation of the spiking network and we quantify the stereo matching performance with a synthetic dataset and an event camera dataset.

## 2. Methods

The stereo-vision architecture introduced here combines two event-based sensors, the Dynamic and Active Pixel Vision Sensor (DAVIS) (Berner et al., 2013), and three VLSI multi-core analog/digital Dynamic Neuromorphic Asynchronous Processors (DYNAPs) (Moradi et al., 2018) integrated in a 4-chip board. As a prototype, we designed the interface between sensing and processing on a dedicated Field Programmable Gate Array (FPGA) device (Xilinx Kintex-7 FPGA on the OpalKelly XEM7360).

### 2.1. Event-Based Sensing

As opposed to classical frame-based cameras, event-based sensor encodes information with lower latency and redundancy (Gallego et al., 2019). Inspired by the biological photoreceptors, the neuromorphic pixels operate independently and send out asynchronous events in response to significant changes in illumination using an event-based data protocol Address Event Representation (AER) (Deiss et al., 1998). The polarity of those events encodes increases (ON events) or decreases (OFF events) in illumination. Overall, this results in fast data acquisition with low latency and high temporal resolution. Compared to the original DVS (Lichtsteiner et al., 2008), the DAVIS sensor features a higher spatial resolution (240 × 180) and adds an APS (Active Pixel Sensor) readout.

**Figure 1:**
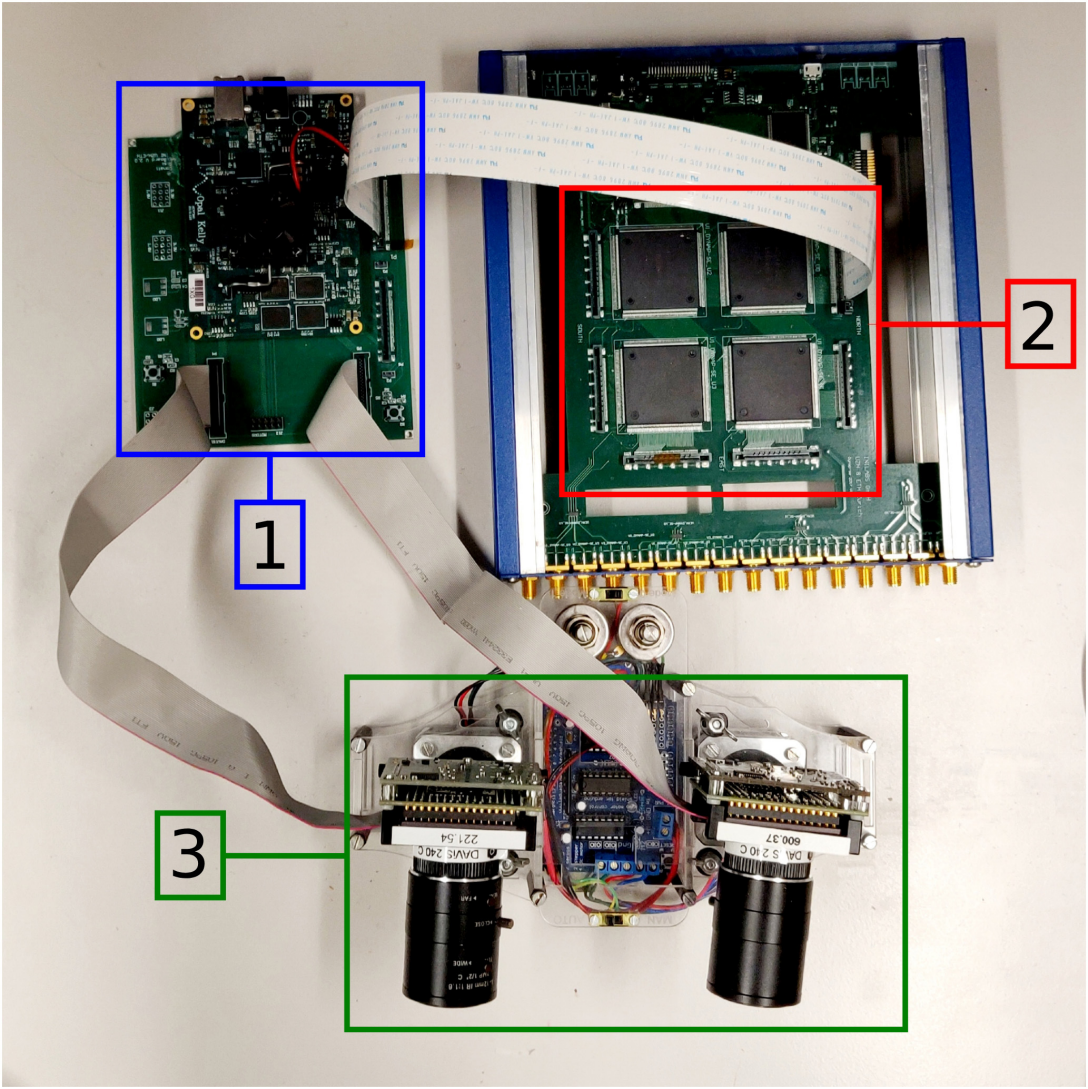
The neuromorphic stereo-vision setup: OpalKelly XEM7360 [1], DYNAP [2], Stereo DAVIS240C [3].

In the proposed architecture, the two DAVIS sensors are mounted on a stereo-setup (see Figure 1) and are separated by a baseline distance of about 6 cm, which is similar to the pupillary distance of humans. Events are sent separately from both retinas to an FPGA using the AER protocol.

### 2.2. Sensors-Processor FPGA Interface

**Figure 2:**
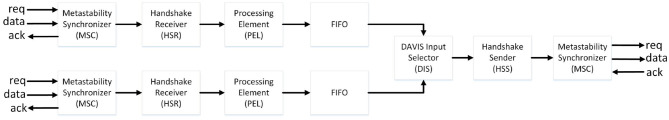
Overview of the event-based digital interface.

Figure 2 shows the main modules of the event-based digital interface. The communication to/from the FPGA is based on a 4-phase handshake protocol, handled by the Handshake Receiver (HSR). Since the 4-phase handshake interfaces two different clock domains, metastable states of the input events could occur. This is handled by the Metastability Synchronizer (MSC) module, which uses a chain of two Flip-Flops to prevent metastability. A pre-processing element (PEL) reduces the input resolution to a 16 × 16 array to redirect the AER events to the destination core on the neuromorphic processor. The pre-processed events are thus forwarded to a small FIFO with eight entries, in charge of absorbing the pipeline stall due to the successive multiplexing stage. The DAVIS Input Selector (DIS) module multiplexes the data using a round-robin scheme and forwards them to the Handshake Sender (HSS), which handles the output handshake with the neuromorphic processor.

### 2.3. Event-Based Processing

The architecture computational substrate is a multi-core asynchronous mixed-signal neuromorphic processor fabricated using standard 0.18 μm 1P6M CMOS technology, the DYNAP (Moradi et al., 2018). Each core comprises 256 adaptive exponential integrate-and-fire (AEI&F) silicon neurons that emulate the biophysics of their biological counterpart, and four different dedicated analog circuits that mimic fast and slow excitatory/inhibitory synapse types (Brette and Gerstner, 2005). Each neuron has a Content Addressable Memory (CAM) block, containing 64 programmable entries allowing to customize the on-chip connectivity. A fully asynchronous inter-core and inter-chip routing architecture allows flexible connectivity with microsecond precision under heavy systems loads. Digital peripheral asynchronous input/output logic circuits are used to receive and transmit spikes via an AER communication protocol, analogous to the one used for the event-based input stream. As a result, the proposed implementation leads to a prototype for a fully asynchronous pipeline of event-based stereo vision.

### 2.4. The Spiking Neural Network Model

**Figure 3:**
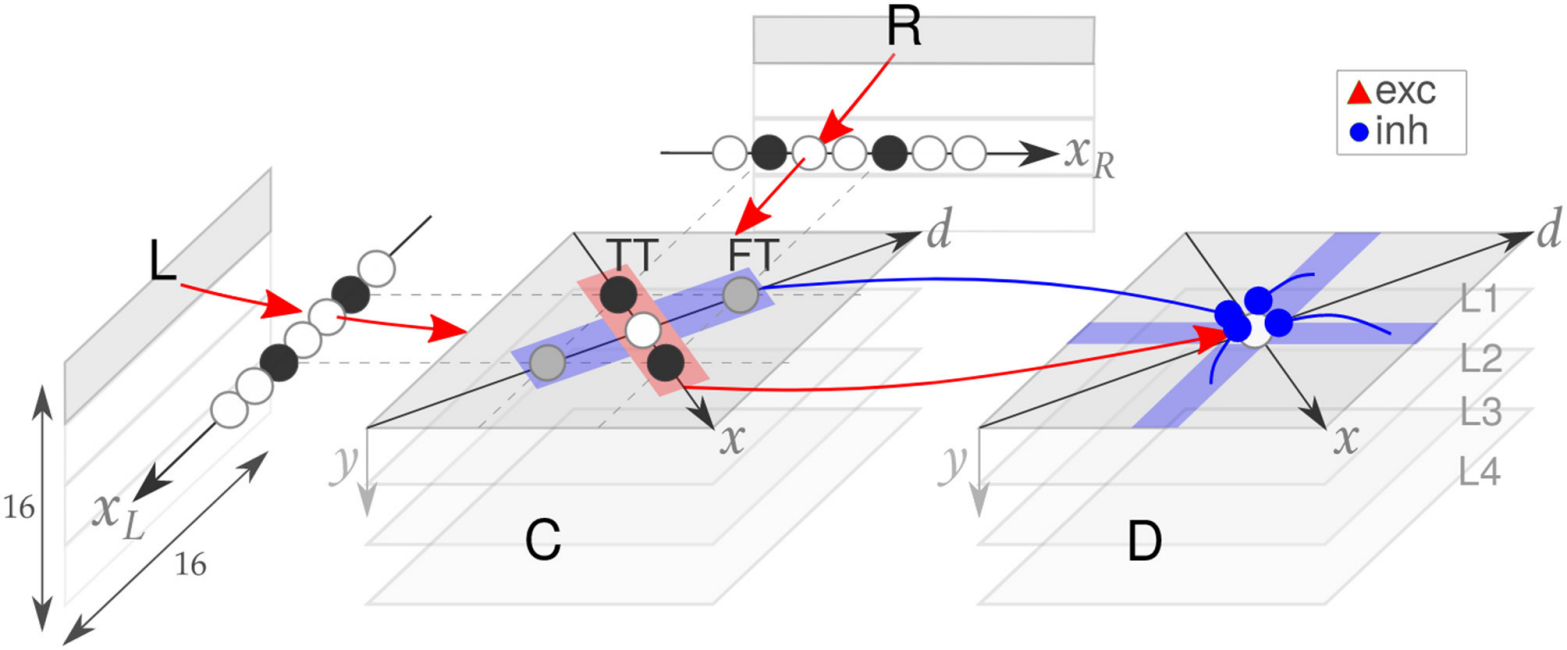
The spiking neural network model. The input space of the retina (R, L) is downscaled and processed by four populations of coincidence (C) and disparity (D) neurons (first one highlighted in light gray). Excitatory (red) and inhibitory (blue) connections are shown. Adapted from Osswald et al. (2017).

The SNN implemented on the DYNAP is adapted from the structure presented in Osswald et al. (2017). It consists of three neuronal populations: the retina, the coincidence detectors, and the disparity detectors (see Figure 3). Each coincidence and disparity neuron is assigned a triplet of coordinates, a horizontal cyclopean position (*x* = *x*_*R*_ + *x*_*L*_), a vertical cyclopean position (*y*), and a disparity value (*d* = *x*_*R*_ − *x*_*L*_), which determines the neuron representation of a location in the 3D space.

Each coincidence neuron receives excitatory inputs from a pair of retina cells tuned to its same spatial location (*x*_*R*_ or *x*_*L*_), thereby encoding temporal coincidences among pairs of inter-ocular events. However, the temporal information is crucial but not sufficient to correctly solve the stereo ambiguity, which arises from matching features from different stimuli. For instance, two stimuli moving synchronously on a plane yield four clusters of activation in the coincidence detectors population: two correct matches along the direction of constant disparity, here referred to as True Targets (TT) and two wrong matches along the direction of constant cyclopean position, here referred to as False Targets (FT), which correspond to the erroneous perception of two stimuli moving in depth.

This ambiguity is reduced in the disparity population by means of two mechanisms of inhibition: recurrent inhibition (Type I) across disparity neurons tuned to the same line of sight (i.e., *x* = *x*_*L*_* or x* = *x*_*R*_) and feed-forward inhibition (Type II) from coincidence neurons tuned to the same cyclopean position. Moreover, disparity neurons receive feed-forward lateral excitation from coincidence neurons tuned to the same disparity. This excitatory-inhibitory balance allows integrating the stimulus spatiotemporal features over time, thereby implementing the matching constraints of cooperative algorithms (Marr and Poggio, 1976; Mahowald, 1994b; Osswald et al., 2017). As a result, the SNN model can solve the stereo matching problem, with only TT represented in the disparity population.

### 2.5. Neuromorphic Hardware Implementation

The entire pipeline of visual information processing was designed to be a scalable neuromorphic architecture. In our proof-of-concept mixed-signal implementation of stereo vision, both coincidence and disparity detectors are implemented using silicon neurons. All neurons in the architecture are emulated by parallel physical circuits in real-time on the neuromorphic processor. In order to optimize the trade-off between the retina field of view and the computational resources on hardware, the input pixels from the event cameras are downscaled to two 2D arrays of 16 × 16 neurons on FPGA which, in turn, project to a 3D array of coincidence detectors. Therefore, the array has a width of 16 neurons, both in the *x*_*R*_ and *x*_*L*_ dimensions. The *y* dimension, instead, is further downscaled to four levels, hereafter referred to as network “layers” L1–4 (Figure 3). The same structure is implemented for the 3D array of disparity neurons. In total, the architecture comprises *N*_*n*_ = 3, 072 silicon neurons and *N*_*s*_ = 62, 562 silicon synapses (see Supplementary Data 1.2 for the estimated power consumption of the network).

#### 2.5.1. Coincidence Detection

**Figure 4:**
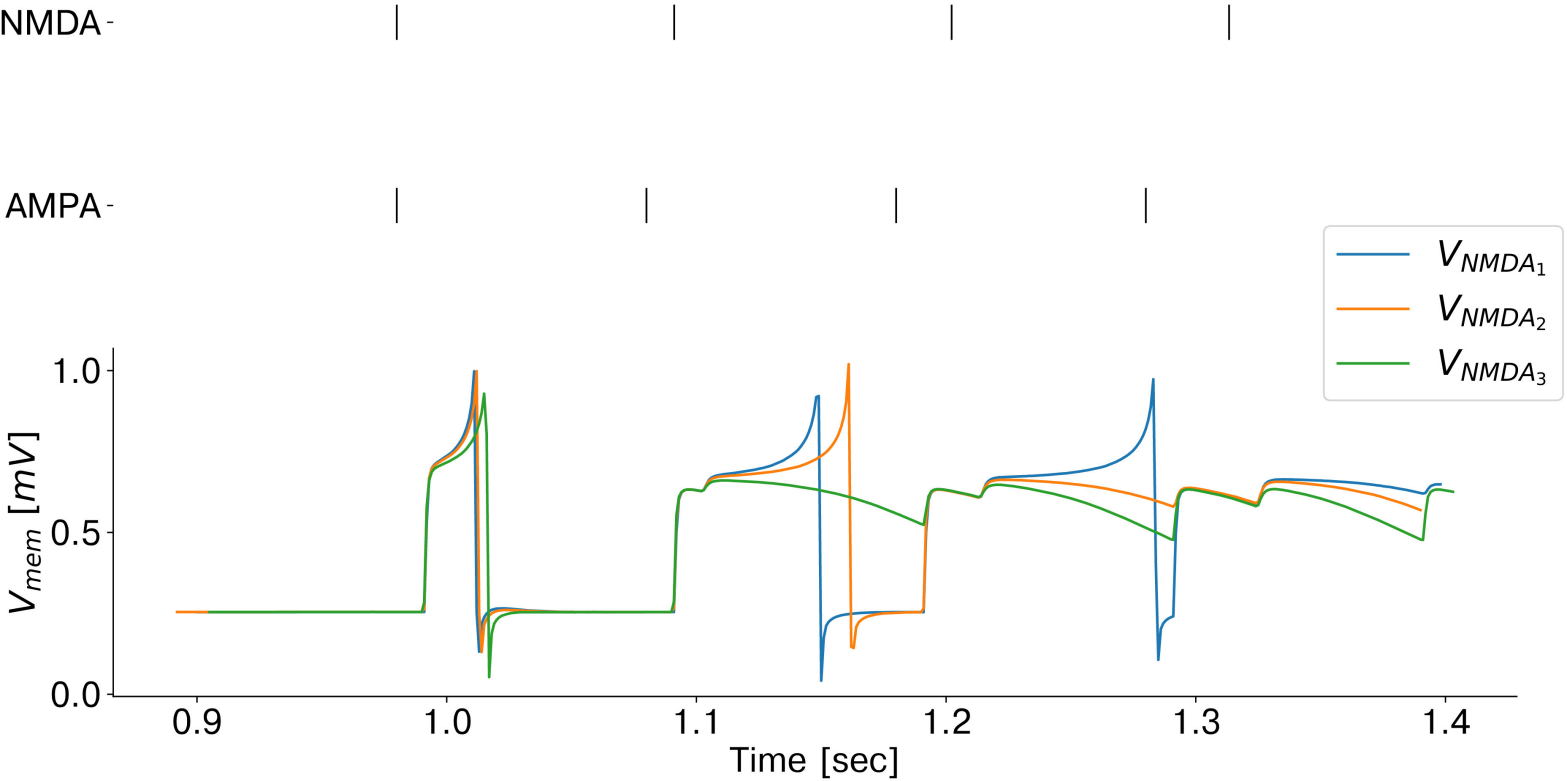
Emulation of coincidence detection: recorded membrane potential of a coincidence detector as a function of the NMDA voltage-gating threshold (*V*_*NMDA*_). As the threshold decreases, the silicon neuron responds to larger inter stimulus interval delays and therefore, the coincidence detector sensitivity increases.

Since coincidence detection is a key component of our model, we carefully emulated and further optimized the low-power mechanism exploited by biological brains. Specifically, temporal coincidences are detected by combining the mechanism of supra-linear, dendritic summation of synaptic events with slow and fast synaptic time constants. As in biological brains, AMPA synaptic currents can boost the effect of slow NMDA synapses when both synaptic inputs are close in time (González, 2011). Coincidence detectors are emulated on the chip exploiting the non-linear properties of the dedicated analog synapse circuit block, which mimics the biological NMDA voltage-gating dynamics. Each coincidence detector is connected to one of the corresponding input retina cells via the slow (NMDA-like) synapse and to the other one via the fast (AMPA-like) synapse circuit block. Only if both synapses are stimulated in rapid succession the coincidence detector neuron fires. A demonstration of coincidence detection emulated on-chip is shown in Figure 4 (see Supplementary Data 1.1 for a full characterization of the proposed coincidence detection building block). To reduce the effect of high-frequency homolateral excitation (Dikov et al., 2017), we included one inhibitory connection from each input neuron to the coincidence detectors population. By controlling the ratio between excitatory/inhibitory synaptic time constants, this helps to suppress incoming monocular events with a high input rate, which would otherwise boost the activation of coincidence detectors, leading to the erroneous perception of inter-ocular coincidences.

#### 2.5.2. Disparity Detection

Lateral feed-forward inhibition was implemented with a separate population of coincidence neurons receiving excitatory connections from the coincidence detectors (Supplementary Figure 3). As a result, the effect of the lateral inhibition is delayed with respect to the feed-forward input from the population of excitatory coincidence detectors. This allows to boost the activity of neurons receiving excitatory inputs due to temporally correlated inter-ocular events and therefore helps to suppress false targets in the disparity population.

#### 2.5.3. Network Calibration

As shown in Osswald et al. (2017), neurons in the emulated SNN model of cooperative stereo vision compute an approximation of the local covariance of the spatiotemporal visual information. As a result, neuronal and synaptic time constants are key parameters in the proposed architecture, and they were configured as follows. First, we measured the distribution of both monocular and interocular inter-spike-intervals of the input events. Then, the time constants of coincidence detectors were set according to the constraints in (Supplementary Data equation S2). Finally, the neuronal time constants of disparity detectors were set significantly larger than the time constants of coincidence detectors, i.e., within the timescale of hundreds of milliseconds.

### 2.6. Experiments

Prior to a full-scale implementation of the prototype architecture, we assessed the stereo matching performance by comparing the network output to an event-based ground truth. We included in our interface design another datapath that uses the OpalKelly USB3.0 to allow high-speed data transfer from the PC. This allowed us to validate the network performance in two scenarios. First, we used a synthetic dataset to test the effectiveness of lateral inhibition with temporally correlated input events. Next, we tested the network performance with real events collected with the event cameras.

#### 2.6.1. Stereo Matching With Synthetic Inputs

**Figure 5:**
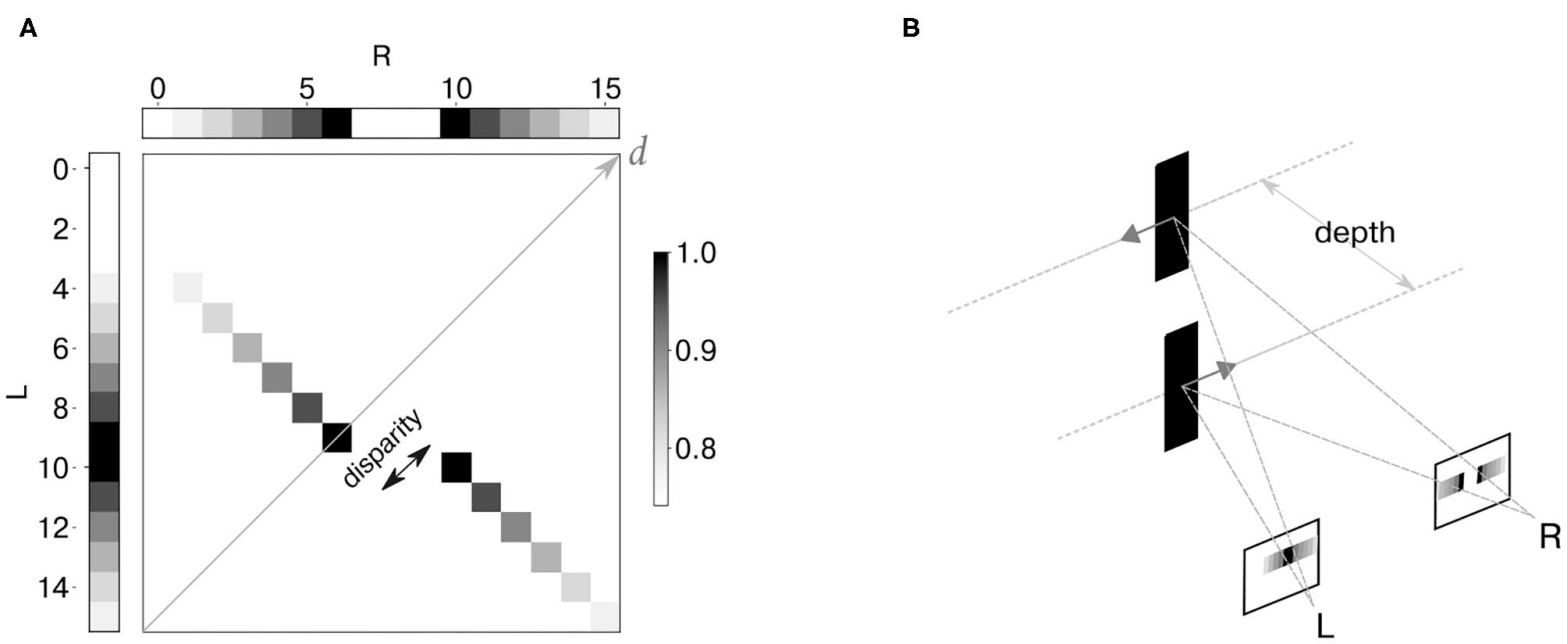
Synthetic dataset: the neural activity is depicted as a temporal image, with gray levels representing synchronous activation in time **(A)**. Two spike trains were generated to simulate the activity of event cameras in response to two edges moving in opposite directions at constant depth levels **(B)**. Input binocular time series and expected activation over time of disparity neurons are shown as temporal images.

As a first step, we generated a synthetic dataset to mimic the output of two neuromorphic retinae recording the scenario of motion on a plane, and specifically two stimuli (dark edges) moving in opposite directions on different depth planes (Figure 5B). The spiking network model in Osswald et al. (2017) is designed to have individual coincidence detectors for each event polarity. However, since a full-scale implementation of the model is out of the scope of this work, we chose to focus our analysis on one event polarity. Figure 5A shows the reproduced activity in the input neurons, together with the expected output of the disparity population. The neural activity is depicted as a temporal image, with gray levels representing synchronous activation in time.

We define as “stimulus speed” the number of input neurons sequentially activated by the stimulus over time. Thus, we chose a speed of 20 input neurons/s for both stimuli, with each input neuron firing at 50 Hz when the stimulus moves to its corresponding location (Supplementary Figure 7A). Moreover, events were generated with vertical coordinates such that they would target only one out of four network layers.

Since the goal is to validate the effect of lateral inhibition, we explicitly constructed the input events with perfect temporal inter-ocular correlation. In this scenario, only if the network uses the lateral inhibition to integrate not only temporal but also spatial features of the stimuli, the ambiguity can be resolved.

#### 2.6.2. Stereo Matching With Event Cameras Inputs

**Figure 6:**
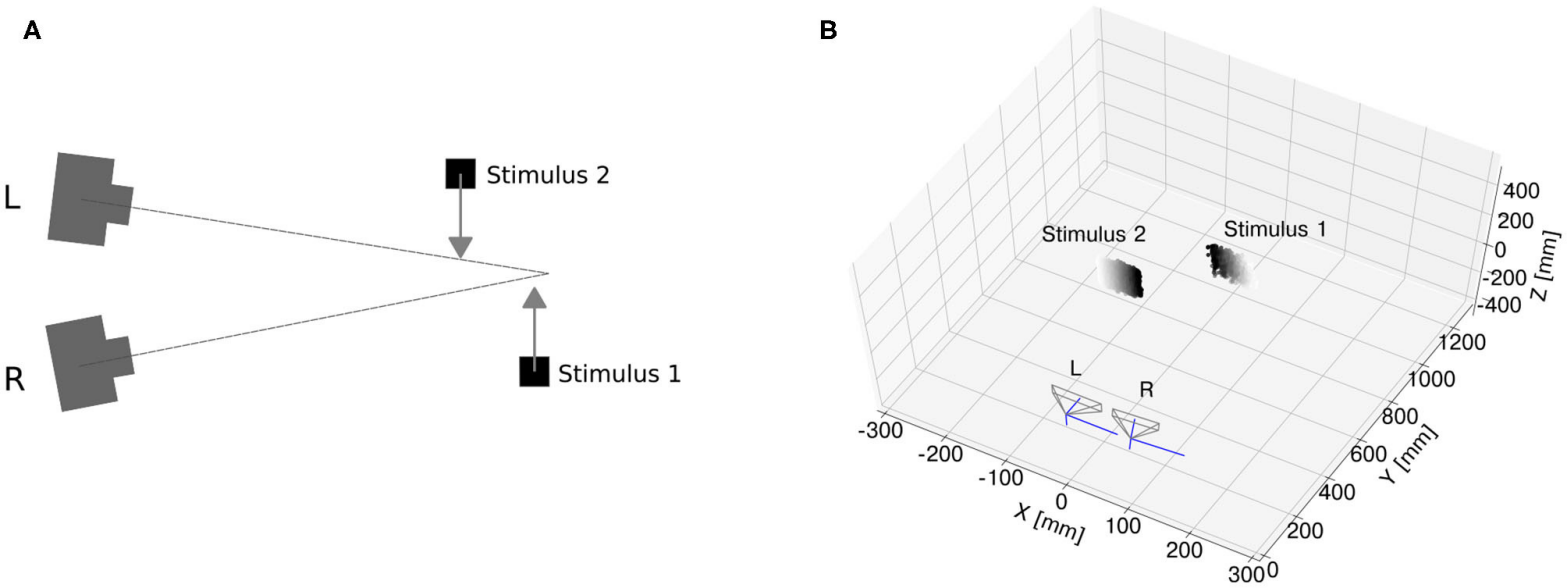
Event camera dataset. Sketch of experimental setup **(A)** two monitors were used for the generation of two edges separated in depth and moving on a plane. After calibration, the monitor generating Stimulus 1 was placed closer to the region of the camera vergence point, while Stimulus 2 was placed closer to the stereo setup. Pointcloud reconstruction **(B)** with generalized time-based technique (time window ϵ = 2 ms, exponential decay kernel τ = 10 ms and a spatial kernel of 10 × 10 pixels).

Real-time scenarios recorded with event cameras inevitably produce noisy events, mainly due to camera jitter and variable latency. Therefore, in order to validate the proposed approach for an end-to-end event-based architecture of stereo vision, it is essential to assess whether the network can still resolve the ambiguity of stereo correspondences with noisy inputs. To this end, we reproduced the scenario of motion on a plane simulated with synthetic data and recorded events from the event cameras. The experimental setup is illustrated in Figure 6A.

The software “Processing” (Reas and Fry, 2007) was used to simulate two dark edges moving on a white background at a constant speed on two different screens. The setup was calibrated using the MATLAB Stereo Camera Calibrator Toolbox with the grayscale images of the DAVIS240C. Upon estimating the camera extrinsics and intrinsics, one screen was placed around the camera vergence point and the second one between the vergence point and the stereo setup. In order to optimize the ratio between spatial resolution and the number of input neurons, a window of 96 × 96 pixels centered around the stimulus was applied to filter out information outside the region of interest, and the recorded events were further downscaled with a kernel of 6 × 6 pixels.

### 2.7. Stereo Matching Performance

#### 2.7.1. Event-Based Ground Truth

In order to assess the stereo matching performance of the network, an event-based ground truth is required. While this is intrinsically available in the case of synthetic datasets, it is not as straightforward with a real dataset. For this scenario, we assumed as true matches the stereo correspondences detected with generalized time-based technique (Ieng et al., 2018) with spatial, temporal, and motion consistency used as matching constraints^1^. To increase the ground-truth accuracy, we fed the generalized time-based technique with one stimulus at a time so that there was no stereo ambiguity. Finally, detected stereo correspondences were labeled as true targets if yielding a correlation score larger than *c* = 0.4 (resulting pointcloud reconstruction shown in Figure 6B).

#### 2.7.2. Accuracy

The stereo matching accuracy was measured with the following metrics proposed in Osswald et al. (2017).

1. Percentage of Correct Matches (PCM):
  (1) $$\begin{array}{l} PCM_{C,D}\left(t_{i}\right)=\frac{TT_{C,D}\left(t_{i}\right)}{FT_{C,D\left(t_{i}\right)}+TT_{C,D}\left(t_{i}\right)} \end{array}$$
  with *TT*_*C,D*_(*t*_*i*_), and *FT*_*C,D*_(*t*_*i*_) being the normalized number of true targets and false targets recorded within a time window *t*_*i*_, both for coincidence and disparity neurons. Spikes were labeled as true targets if the minimum euclidean distance in the 2D plane (*x*, *d*) between the recorded neuron id and the ground truth neuron ids was smaller than the threshold distance *D*_*min*_ = 1.
2. True Target Amplification (TTA) and False Target Amplification (FTA):
  (2) $$\begin{array}{l} TTA=\frac{\sum_{t_{i}}TT_{D}\left(t_{i}\right)}{\sum_{t_{i}}TT_{C}\left(t_{i}\right)}\;FTA=\frac{\sum_{t_{i}}FT_{D}\left(t_{i}\right)}{\sum_{t_{i}}FT_{C}\left(t_{i}\right)} \end{array}$$
  which allow quantifying the disparity sensitivity (TTA) and the degree to which false targets are suppressed due to recurrent and lateral feed-forward inhibition (FTA).

## 3. Results

### 3.1. Stereo Matching With Synthetic Inputs

**Figure 7:**
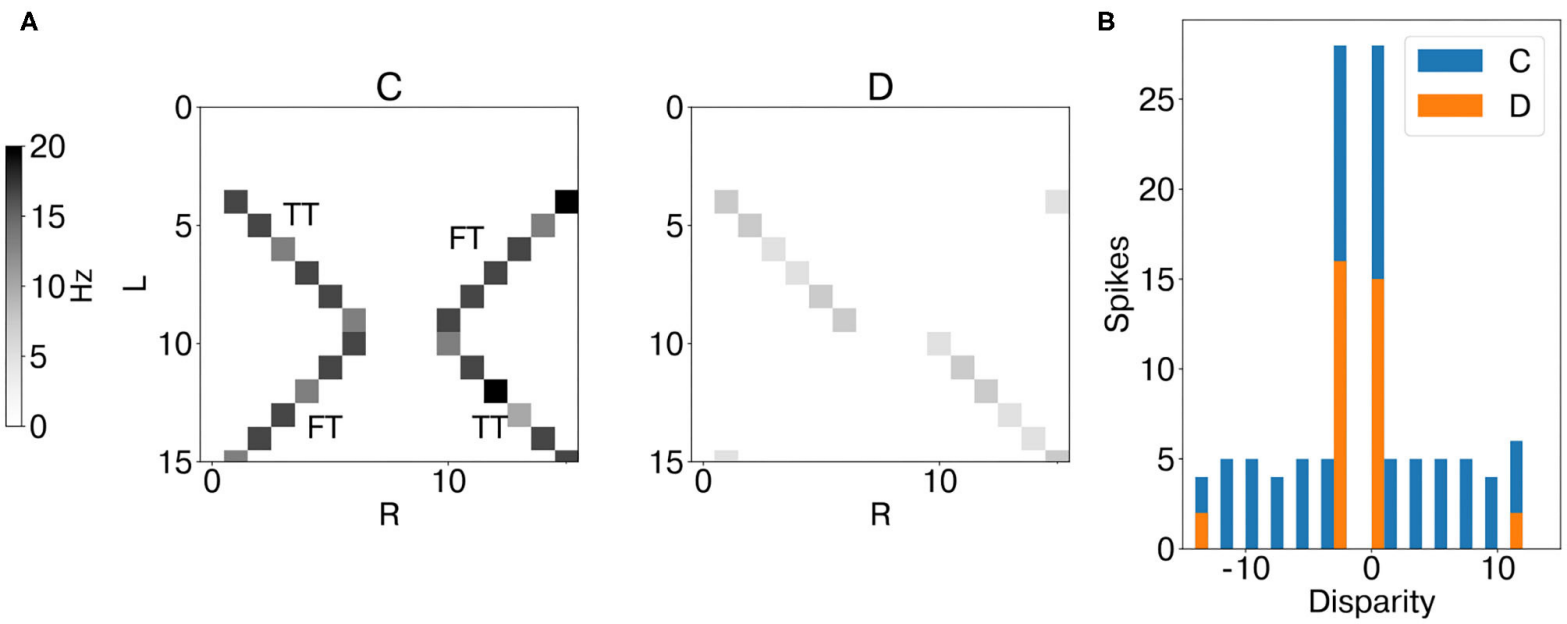
Results of network emulation with the synthetic dataset. Mean firing rate of coincidence neurons (C, excitatory population) and disparity population (D) **(A)**. Histogram of encoded disparity values across the trial duration in both coincidence (blue) and disparity neurons (orange) **(B)**. As the activity clusters around the true disparity values (d = 0, d = −3), the disparity population successfully resolves the stereo ambiguity.

Figure 7 shows the mean firing rate of coincidence (excitatory population) and disparity neurons during the whole trial. The coincidence detectors successfully detect the temporal matches, i.e., an action potential arises only when the input events from the retina cells are coincident in time. However, coincidence detectors still respond to false targets, i.e., coincident events arising from different stimuli. Indeed, in this scenario, binocular time series related to different stimuli are perfectly synchronized (Supplementary Figure 8A) and therefore not distinguishable from the true targets in the temporal domain (Mulansky and Kreuz, 2016). However, as they activate coincidence detectors along the dimension of constant cyclopean position, they also trigger the activation of the correspondent inhibitory coincidence detectors, leading to inhibition of disparity detectors tuned to the same cyclopean position (Supplementary Figure 4). This is not the case for true targets as binocular events due to the same stimulus target coincidence detectors along the dimension of constant disparity, which injects excitatory current into target disparity detectors tuned to the same disparity. As a result, disparity detectors integrate evidence of true disparities and effectively solve the stereo ambiguity.

This is well-depicted by the metrics of stereo matching performance. Compared to coincidence detectors, disparity neurons can successfully suppress false targets (FTA = 0.08), while still being responsive to true targets (TTA = 0.45). This leads to a PCM score of 0.88, compared to PCM = 0.57 for coincidence detectors.

**Figure 8:**
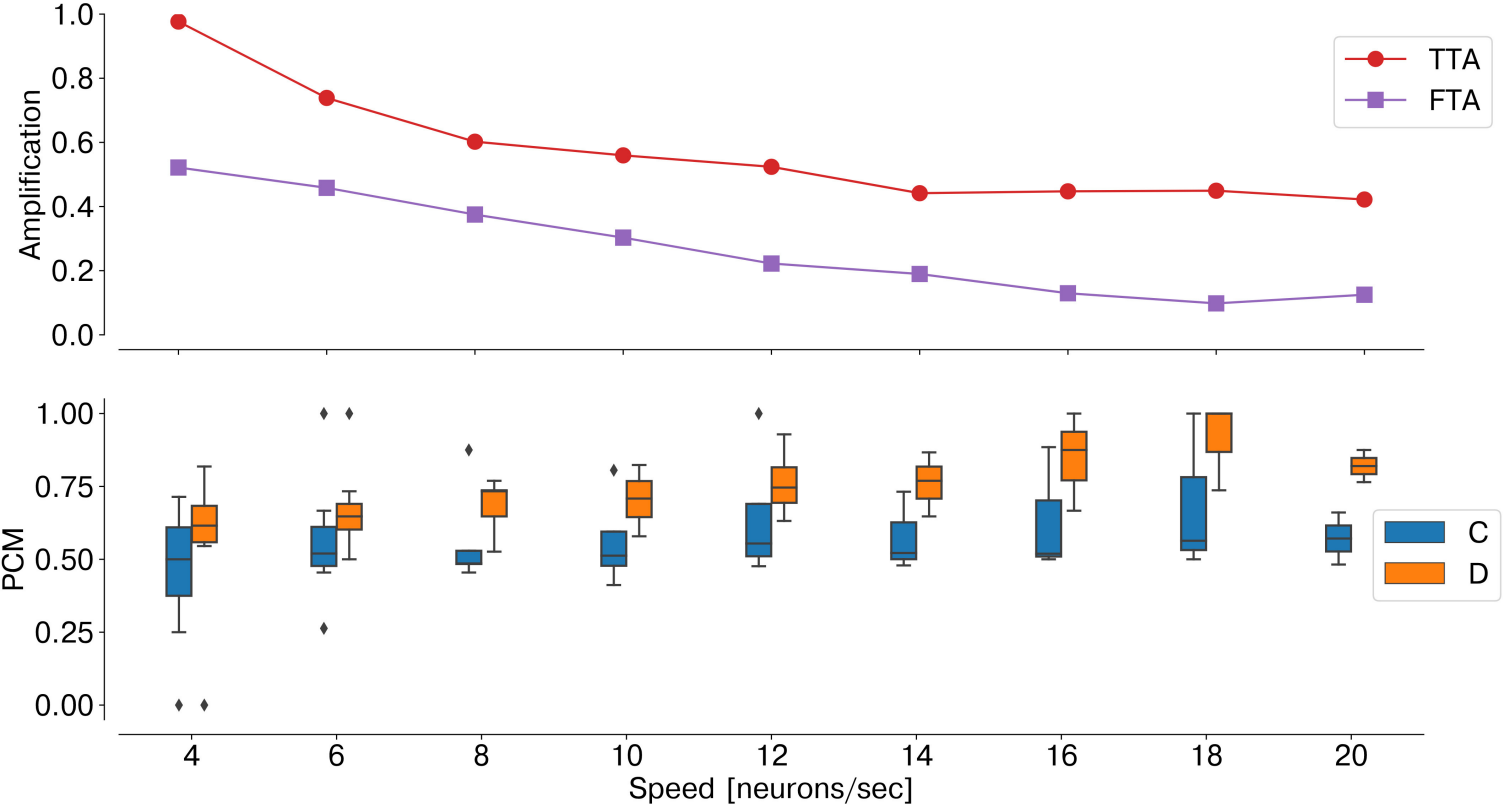
Performance sensitivity to the stimulus speed: PCM (bottom graph, with median and interquartile range, measured across one trial over time windows *t*_*i*_ = 300 ms), TTA, and FTA (top graph). As the stimulus speed increases, the stereo matching performance increases (i.e., lower FTA).

As temporal information is the key feature for an event-based network, the stimulus speed is a crucial factor influencing the network performance. Indeed, as the number of input neurons sequentially activated by the stimulus decreases, the ratio TTA/FTA decreases, thereby affecting the stereo matching performance (Figure 8).

### 3.2. Stereo Matching With Event Cameras Inputs

**Figure 9:**
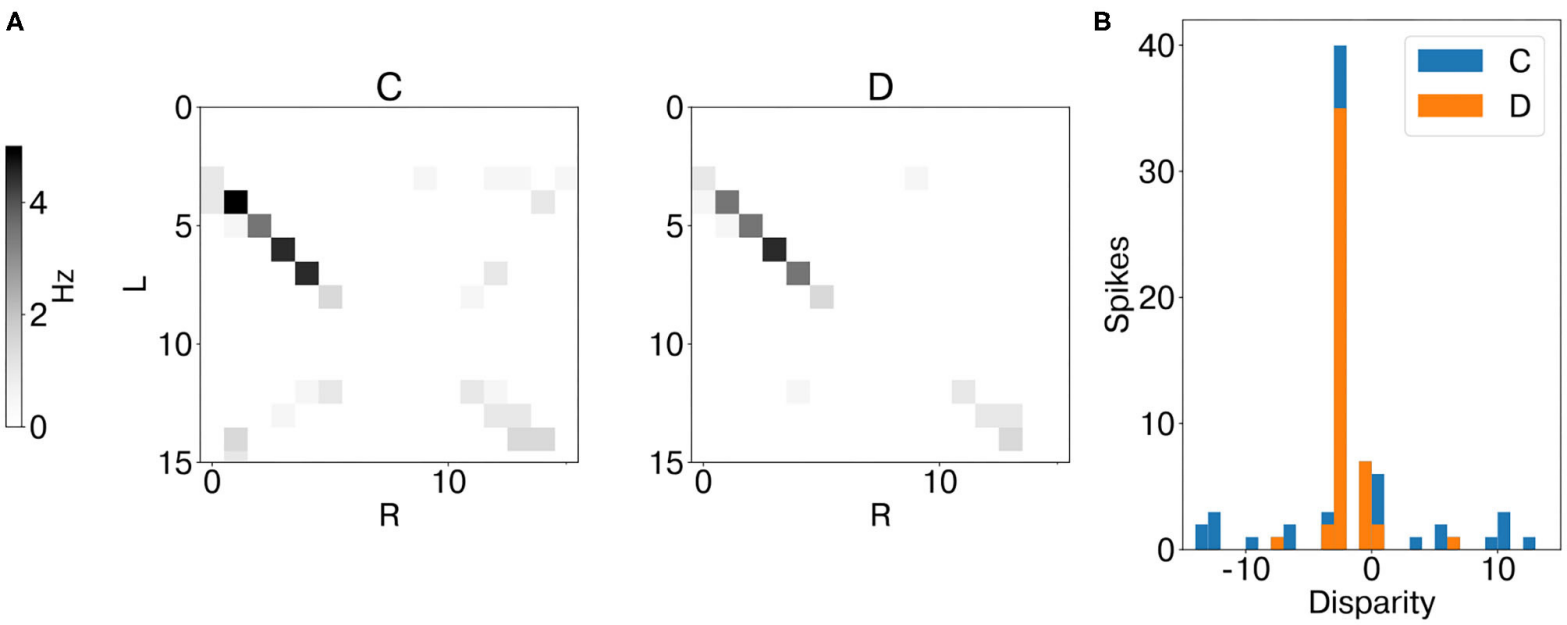
Results of network emulation with event camera dataset from network layer L2. Mean firing rate of coincidence neurons (C, excitatory population) and disparity population (D) **(A)**. Histogram of encoded disparity value across the trial duration in both coincidence (blue) and disparity neurons (orange) **(B)**. As the activity clusters around the true disparity values, the disparity population successfully resolves the stereo ambiguity.

**Figure 10:**
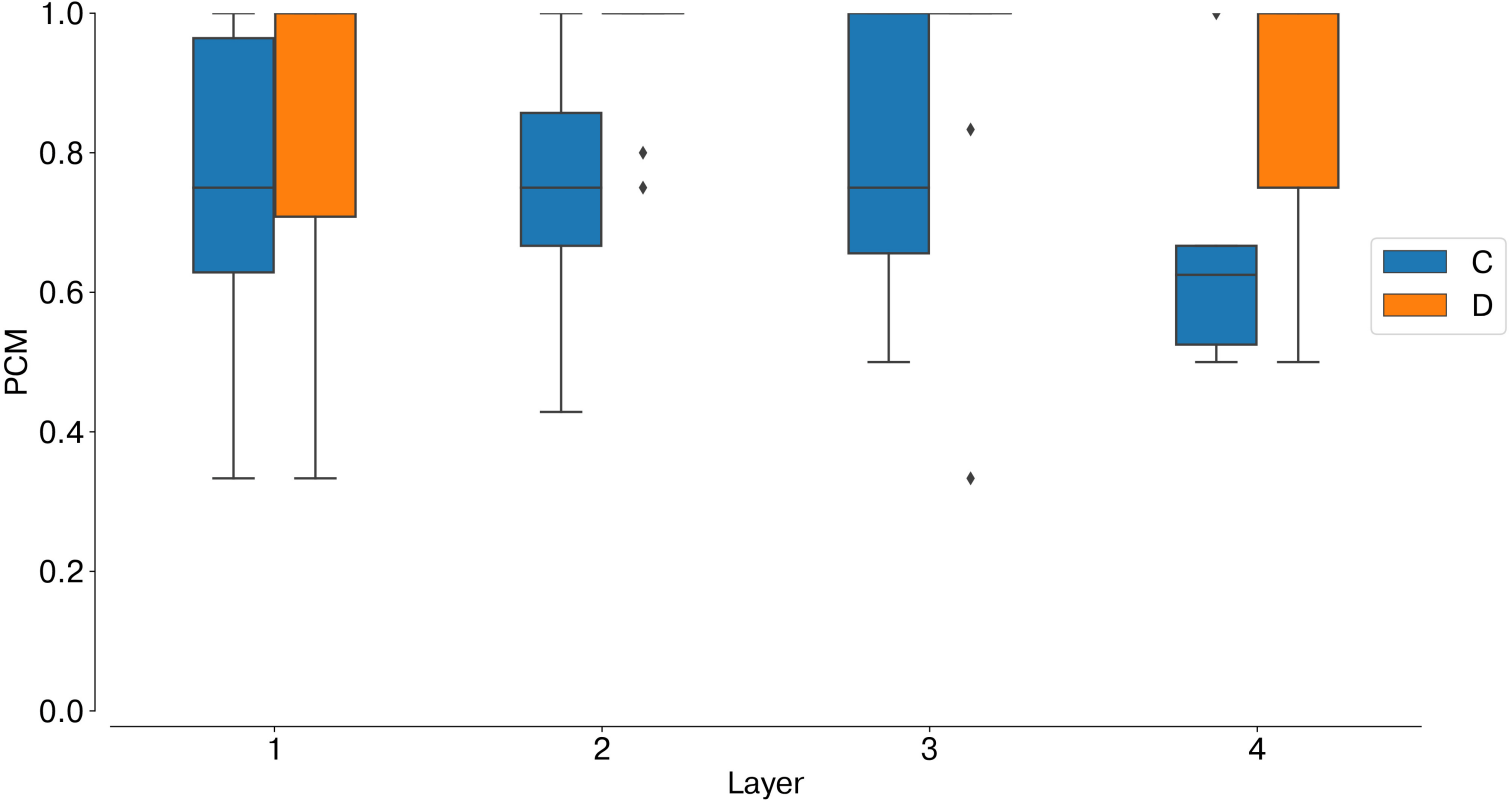
Stereo matching accuracy: PCM, median and interquartile range. In all network layers (L1–4) the PCM of disparity neurons is larger than the PCM of coincidence neurons, showing that disparity detectors can still solve the stereo ambiguity with slower, and uncorrelated, real stimuli.

Analogously to the analysis performed with synthetic data, we first measured the average instantaneous firing rate of coincidence and disparity neurons during one trial with data from the event cameras. Notably, binocular time series of non-correspondent stimuli are less correlated in real scenarios (Supplementary Figure 8B). Therefore, false and true targets become more separable from the temporal information already. This is why the activation of coincidence detectors responding to false targets is reduced compared to those responding to true targets (Figure 9). However, disparity detectors still achieve better performances in resolving the stereo ambiguity (Figure 10).

## 4. Discussion

We have presented a prototype architecture for cooperative stereo vision implemented on a scalable neuromorphic architecture. Recovering the 3D structure of a scene is still computationally expensive for conventional computer vision approaches. Yet, biology shows several examples of stereo vision whereby space-variant and asynchronous space-time sampling are some of the key features involved. With parallel, sparse, and asynchronous computation, neuromorphic hardware promises to offer an optimal substrate for a low-latency implementation of 3D vision. However, only a few approaches developed so far fully exploit the advantages of analog asynchronous computation. Hereby we implemented a biologically-inspired, event-based network of stereo vision on a mixed analog-digital neuromorphic processor and we validated the stereo matching performances of the architecture.

Our model is derived from the work of Osswald et al. (2017), which presents software simulations of the full-scale implementation. By solving the stereo-matching problem with leaky-integrate-and-fire neurons, the simulated spiking network proves an effective approach to fully exploiting the event-based visual sensors. However, the full potential of the model and its scalability can only be leveraged if the neurons operate in parallel. Here we validated the stereo-matching abilities of the network by implementing it on a massively parallel neuromorphic processor. Compared to the previous feasibility study based on the ROLLS chip (Osswald et al., 2017), the proposed solution shifts the coincidence detection mechanism, previously on FPGA, directly on analog silicon neurons. Exploiting the non-linear properties of a dedicated analog circuit, that mimics the biological NMDA voltage-gating dynamics, led to a robust coincidence detection mechanism that could ease the network sensitivity to device mismatch, which is a crucial feature of subthreshold mixed-signal neuromorphic processors. In this regard, we anticipate that quantifying the effect of device mismatch on coincidence detection will be a crucial step prior to a full-scale implementation of the network on-chip.

In order to validate the effectiveness of the neuromorphic substrate in solving the stereo correspondence problem, we assessed the network performances in two scenarios. First, with a synthetic dataset, we demonstrated the crucial role of the synaptic kernel of feed-forward lateral inhibition. To do so, we explicitly constructed the input binocular time series such that false targets would be temporally correlated and, therefore, only distinguishable from the true matches if disparity neurons integrated the stimulus spatiotemporal features. However, this is only possible when the temporal dynamics of the stimulus are comparable with the neuron synaptic time constants, as we showed in Figure 8. In other words, as the network exploits motion cues to solve the stereo matching problem, the network temporal sensitivity becomes intrinsically related to the network spatial resolution. Thus, the number of input neurons sequentially activated by the moving stimulus over time is a crucial factor: increasing the number of neurons sensitive to the input field of view would restore the network sensitivity to lower speed stimuli.

The second scenario with data from event cameras allowed us to test the network performance with noisy time series, whereby non-correspondent inter-ocular events are not perfectly correlated. Here the lateral inhibition fails due to lower speed stimuli (Supplementary Figure 7B). Yet the network can still achieve good stereo matching performances due to the recurrent inhibition, which triggers competition among disparity neurons tuned to the same line of sight. In this scenario, the feed-forward excitatory input from coincidence detectors responding to temporally correlated stimuli boosts the activation of disparity neurons responding to true targets, therefore successfully leading to false target suppression again.

Overall, both experiments validate our approach with stimulus motion yielding constant disparity. The future step is testing the network dynamics in the case of motion-in-depth, which naturally addresses the trade-off accuracy vs. speed. Indeed, coincidence detectors feature low-latency response to short inter-ocular time differences, thereby setting the network temporal resolution within the timescale of microseconds. Disparity detectors, by contrast, need to integrate the stimulus motion cues over time to resolve the stereo ambiguity, and therefore they require longer neuronal time constants (up to 100 ms). In fact, by receiving excitatory and inhibitory projections from coincidence and inhibitory neurons, respectively, disparity neurons compare evidence of the current stimulus statistics against the integrated evidence of the stimulus spatiotemporal features. Measuring the network response in the case of motion in depth will allow investigating the effect of this excitatory/inhibitory balance on the stereo-matching performances. Moreover, since no synaptic plasticity is included in the architecture and given the event-based nature of the input stimulus, a prior assumption about the stimulus statistics is currently required to calibrate the network. Future implementations on the new generation of DYNAP chips will allow to incorporate mechanisms of short-term plasticity, thereby enabling an autonomous adaptive calibration procedure.

Although the architecture proposed is scalable by construction, implementing very large-scale systems based on such architecture, able to operate in real-time, requires adequate resources, and supporting neuromorphic processing hardware. The DYNAP processor used in this study comprises only 1,024 neurons, distributed among four cores of 256 neurons each. However, the routing scheme implemented on that device supports all-to-all connections of up to 16 by 16 chips providing already the ability to scale the system up to 256k neurons. This would, however, require very large printed circuit boards, or many boards interconnected among each other. The DYNAP chips proposed in Moradi et al. (2018) could be integrated into a system comprising a much higher number of cores [e.g., the IBM TrueNorth chip has 4,096 cores (Merolla et al., 2014), and the Intel Loihi chip has 128 cores (Davies et al., 2018)] without making any changes to the design. This would enable the construction of larger scale stereo-vision setups that would still be able to operate in real-time, given the parallel processing ability of the emulated neurons and synapses. We anticipate that designing an end-to-end asynchronous dedicated architecture of this type would allow to fully leverage the potential of sparse, event-based computation of SNN models of cooperative stereo-matching. An additional strategy that would enable the construction of large-scale stereo-vision setups would be to use more complex vision pre-processing stages, for example, implemented using convolutional networks and applying the same principles presented in this work to the features extracted by the convolutional network, rather than the raw pixel values. This would allow us to use a smaller feature space compared to the resolution of the vision sensor, and increase robustness to noise in the vision sensors. As discussed in Steffen et al. (2019), although there are many methods for event-based depth estimation, the lack of a comprehensive dataset or a standard testbed makes it difficult to compare them. Yet, some event-based datasets for stereo vision have been recently released (Andreopoulos et al., 2018; Zhu et al., 2018). Implementing the full-scale model on new generations of mixed analog/digital neuromorphic processors would allow comparing the architecture performances against already existing methods. In the long-term, the goal of the approach proposed is to enable on-chip estimation of depth on a per-event basis, with the highest resolution confined around the camera vergence point. Indeed, conventional approaches of event-based stereo vision constrain the search window for stereo matches along the epipolar lines, which results in the point of zero disparity to be shifted at infinity, and depth error increasing quadratically with depth. Instead, in this work, we took inspiration from the biological coarse and space-variant sampling and processed the raw events with large input search zones. In other words, here disparity detectors tuned to zero disparity respond to targets moving around the camera vergence point. While this naturally constrains the spatial (and therefore depth) resolution, it could set out an optimized solution with latency response and space-variant sampling. Combined with vergence control, this active perception strategy could lead to promising solutions for embedded neuromorphic architectures of stereo vision in humanoid robots (Gallego et al., 2019). Moreover, the need for compelling benchmarks that could show the advantages of spike-based computation in real-world scenarios is currently one of the major challenges for the neuromorphic research field (Davies, 2019). Our solution could show a valuable example of exploiting spike-timing to process real-time information in closed-loop systems, by emulating sparse, parallel computation of biological neurons in order to solve the stereo matching problem.

## Data Availability Statement

The raw data supporting the conclusions of this article will be made available by the authors, without undue reservation.

## Author Contributions

NR did the research and wrote the manuscript. AA designed the digital interface. ED designed the interface between the neuromorphic chip and the OpalKelly board. ED, SS, and GI supervised the work. All authors contributed to the article and approved the submitted version.

## Conflict of Interest

The authors declare that the research was conducted in the absence of any commercial or financial relationships that could be construed as a potential conflict of interest.

## Footnotes

^1^As the DAVIS240C does not integrate the synchronous luminance information, the luminance consistency constraint could not be included in our analysis.
