# Supplementary material for: A Spike-Based Neuromorphic Architecture of Stereo Vision
Source: Front Neurorobot. 2020 Nov 13;14:568283. doi: 10.3389/fnbot.2020.568283 (PMC7693562; doi:10.3389/fnbot.2020.568283)
Supplement: Supplementary file 1 [file Data_Sheet_1.PDF]

Supplementary Material

1 SUPPLEMENTARY DATA

1.1 Coincidence Detection

Prior to measuring the network stereo-matching performance, we validated the effectiveness of combining AMPA-like and NMDA-like analog synaptic circuits as a computational building block to implement coincidence detection. To this end, we connected a population of N coincidence detectors to a pair of input excitatory neurons via the two types of DYNAP Differential Pair Integrator (DPI) synaptic circuits (Chicca et al., 2014), which mimic AMPA and NMDA synapses (Figure S2A). We exploit the voltage-gating mechanism of the NMDA-like DPI synapse to increase the coincidence detectors sensitivity, which is controlled by the DPI threshold V_{NMDA}. The higher the threshold, compared to the membrane voltage of the silicon neuron V_{mem}, the lower the amplitude of the excitatory post synaptic current (EPSC). In order to mimic the biological non-linear summation of synaptic events with slow and fast time synaptic time constants, we set the AMPA and the NMDA synapse time constants to be respectively below and above the inter-stimulus-interval (ISI) (Figure S1). Finally, we defined a coincidence score C that measures the

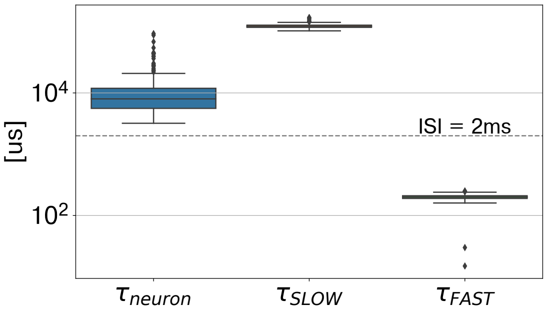

Figure S1. Coincidence detection experiment: time constant distribution set for the analog silicon neurons (τ_{neuron}) and synapses (τ_{SLOW}, τ_{FAST}), shown with median and interquartile range. The time constant of the AMPA-like synapse (τ_{FAST}) is set smaller than the inter-stimulus-interval (ISI=2ms).

effectiveness of our implementation as follows:

$$C = \frac{1}{N}\sum_{i=1}^{N}\left[\tilde{r}^i_{AB} - 2\cdot(\tilde{r}^i_A + \tilde{r}^i_B)\right], \quad \text{with } \tilde{r}_{AB,A,B} = \begin{cases} 1, & \text{if } r_{AB,A,B} > 0 \\ 0, & \text{otherwise} \end{cases} \tag{S1}$$

where r_{AB} is the spike-count rate in response to input pair A+B, and r_A, r_B are the spike-count rate in response to one spike only (A or B). Thus, the closer the coincidence score is to 1, the larger the fraction of analog coincidence detectors responding to the input pair only, and therefore the better the circuit performs as a coincidence detection building block.

Figure S2B illustrates the coincidence detection score measured in four different scenarios, resulting from the combinations of two factors: the NMDA-like DPI threshold V$_{NMDA}$ (columns) and the order of the synaptic events (AMPA-NMDA or NMDA-AMPA, rows). In all four conditions, the coincidence score is reported as a function of the synaptic weights (w_{FAST} of the AMPA DPI and w_{NMDA} of the NMDA

DPI). Notably, for large weights w_{SLOW}, the configuration with low V_{NMDA} threshold leads to a negative coincidence detection score, while the high V_{NMDA} scenario leads scores close to 1. This confirms that the thresholding mechanism of the slow NMDA DPI synapse, with large V_{NMDA}, combined with the AMPA DPI synapse, can effectively implement coincidence detection. In this regard, combining the two different synaptic temporal dynamics, that mimic the biological timescales, might play an important role. Further experiments in this direction should explicitly quantify the effect of synaptic time constants on the coincidence score. However, these results allowed us to define the following constraints to calibrate the network parameters:

$$\tau_{FAST} < ISI_{bin} < \tau_{neuron} < \tau_{SLOW} < ISI_{mon} \quad \text{(S2)}$$

with ISI_{bin} being the average binocular inter-spike-interval and the ISI_{mon} being the average monocular inter-spike-interval. Finally, despite the asymmetric connectivity due to the two different synaptic circuits involved, the reported coincidence score is invariant with respect to the order of synaptic events, yielding a symmetric circuit response.

Upon validating the proposed implementation as an effective candidate for asynchronous, low-power coincidence detection, we incorporated this building block in our architecture for stereo vision (Figure S3).

1.2 Power Consumption

The power consumption of a silicon neuron n on the DYNAP architecture, including spike generation and routing as primitive operations, can be estimated as follows:

$$P_n = r_{inp}\big(E_{spike} + E_{pulse}\big) + r_{out}\big(E_{en} + E_{br} + RT \cdot E_{rt}\big) \quad \text{(S3)}$$

where:

r_{inp}, r_{out} = mean input and output firing rate
E_{spike} = 883 pJ, energy to generate one spike
E_{pulse} = 324 pJ, energy of the pulse extender circuit
E_{en} = 883 pJ, energy to encode one spike and append destination
E_{br} = 6.84 nJ, energy to broadcast event to same core
E_{rt} = 360 pJ, energy to route event to different core
RT = 1, if the spike is sent to a different core, zero otherwise

Given the $N_{pop} = 3$ populations implemented on the neuromorphic processor, and pooling across all neurons N_p in each of them, the total estimated power consumption is:

$$P = \sum_{p=1}^{N_{pop}} \sum_{n=1}^{N_p} P_n^p = \sum_{p=1}^{N_{pop}} \Big[\sum_{i=1}^{N_{inp,p}} r^p_{inp[i]}(E_{spike} + E_{pulse}) + \sum_{j=1}^{N_{out,p}} r^p_{out[j]}\big(E_{en} + E_{br} + RT^{p,j} \cdot E_{rt}\big)\Big] \quad \text{(S4)}$$

where:

r^p_{inp}, r^p_{out} = mean firing rate of input and output populations
$N_{inp,p}$ = number of populations targeting population p
$N_{out,p}$ = number of populations targeted by population p
$RT^{p,j}$ = 1, if populations p and j are implemented on different cores

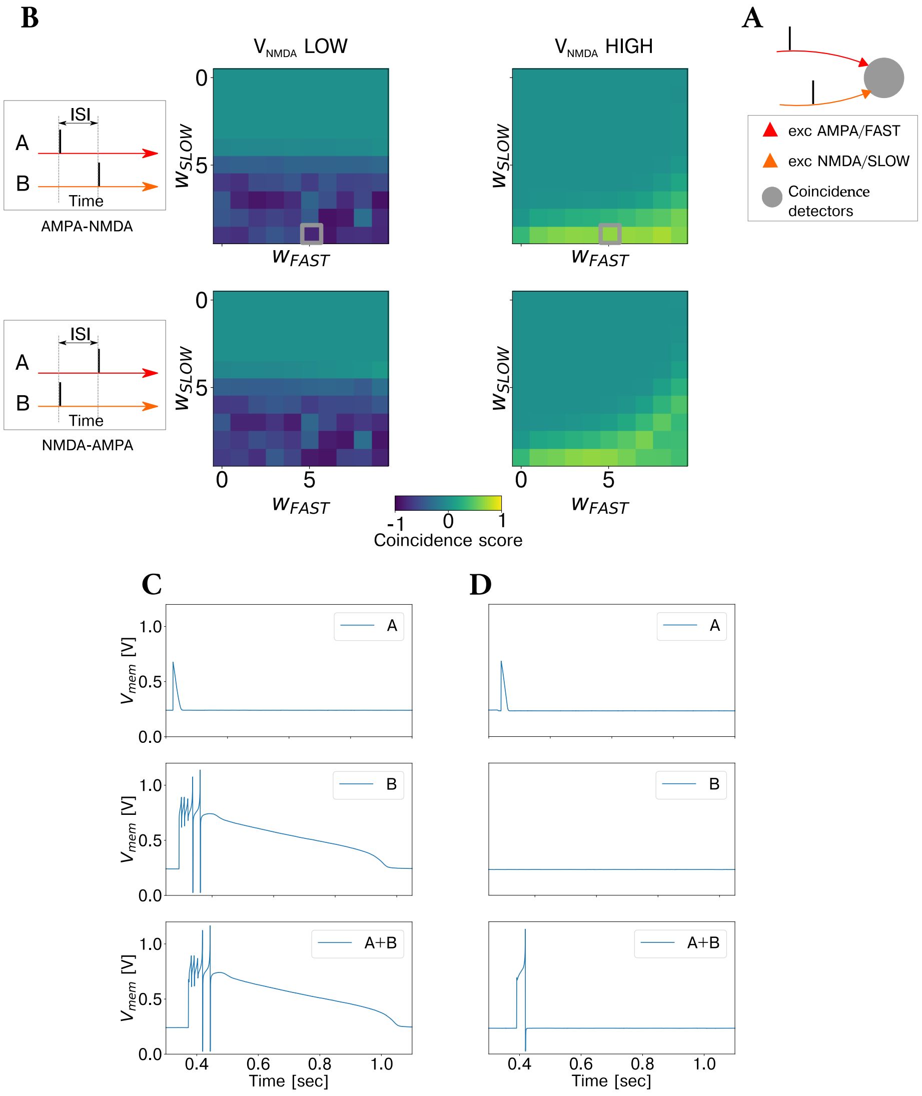

Figure S2. Coincidence detection experiment: network schematic (**A**), coincidence detection score (**B**), and example voltage traces (V_{mem}) recorded during the AMPA-NMDA experimental protocol (w_{FAST} and w_{SLOW} highlighted in **B**), with V_{NMDA} low (**C**) and high (**D**). Only the second scenario leads to an effective building block for coincidence detection, with one spike elicited only when both input spikes are present (A+B vs A or B only). This is because the voltage-gating mechanism of the NMDA DPI, combined with a large V_{NMDA}, prevents the EPSC from rising if there is no previous synaptic event boosting the membrane voltage V_{mem}.

The estimated power consumption in our architecture, for the event-camera dataset, is P=$8.1\,\mu$W. This result refers to the $180\,\mathrm{nm}$ CMOS technology of the DYNAP chip. Using more advanced technology, such as the $28\,\mathrm{nm}$ process, would significantly reduce the power consumption per primitive operation. An estimate for the full network in such scenario is reported in (Osswald et al., 2017). Although the potential of this technology is not yet fully exploited in the current architecture, due to the prototype digital interface between sensing and processing, our results set the stage for designing a new low-power, end-to-end asynchronous, dedicated architecture for stereo vision.

2 SUPPLEMENTARY FIGURES

2.1 Figures

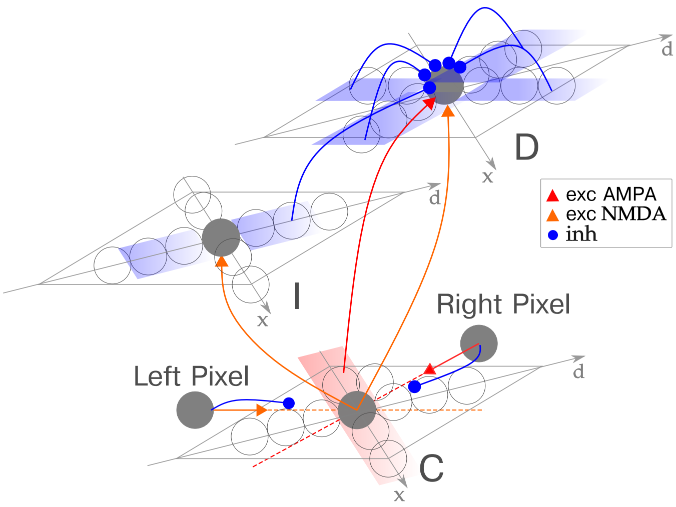

Figure S3. Network connectivity kernels. Detailed view of the synaptic kernels for one cross-section of the network along the vertical dimension: coincidence detectors, excitatory neurons (C), coincidence detectors, inhibitory neurons (I), and disparity neurons (D).

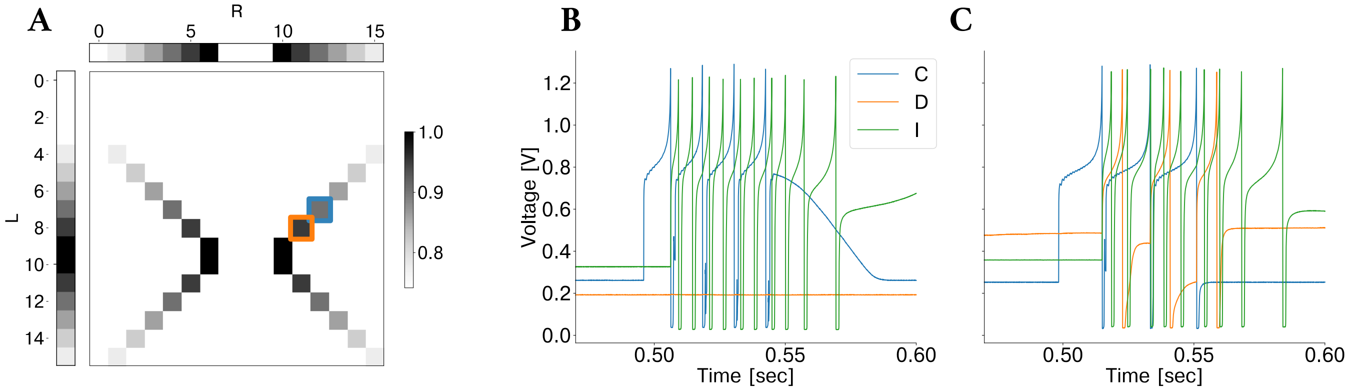

Figure S4. Effect of feed-forward lateral inhibition with fast-moving stimuli. The temporal image **(A)** shows how the neural activation in the coincidence layer moves over time in response to the input stimuli. Highlighted squares show the position of three monitored silicon neurons tuned to the same cyclopean position (and to the same cyclopean position for coincidence and inhibitory neurons). Real-time membrane voltage measured from three silicon neurons **(B-C)**. As the stimulus moves, it activates first the coincidence neuron ($t < 0.5$ sec). Due to the feed-forward lateral inhibition along the direction of constant cyclopean position, this results in inhibitory current injected into the recorded disparity neuron as the inhibitory neuron activates ($t > 0.5$ sec). Therefore, when the excitatory feedforward input from the coincidence layer moves to its corresponding location, the disparity neuron is inhibited (**B**). This is indeed not the case if the lateral inhibition is removed (**C**).

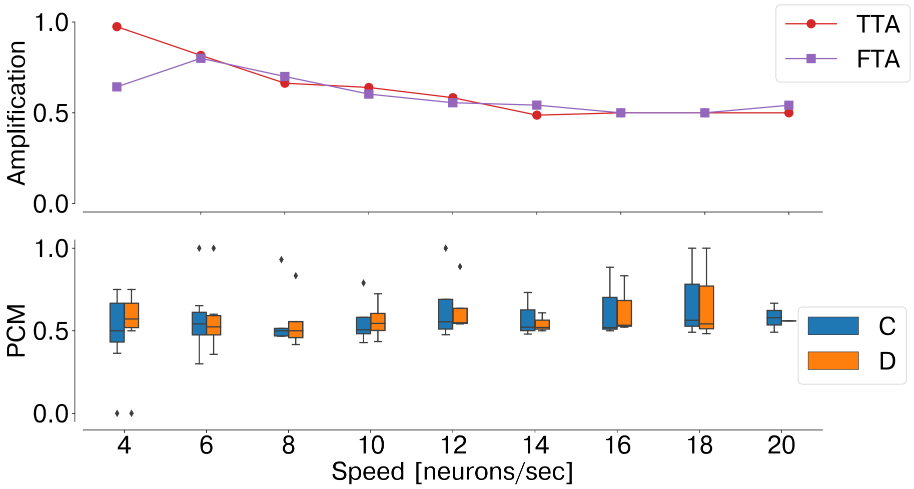

Figure S5. Performance of stereo matching when lateral inhibition is removed. As the lateral inhibition is crucial in suppressing false targets with high-speed, temporally correlated input stimuli, removing lateral inhibition results into a stereomatching performance drop.

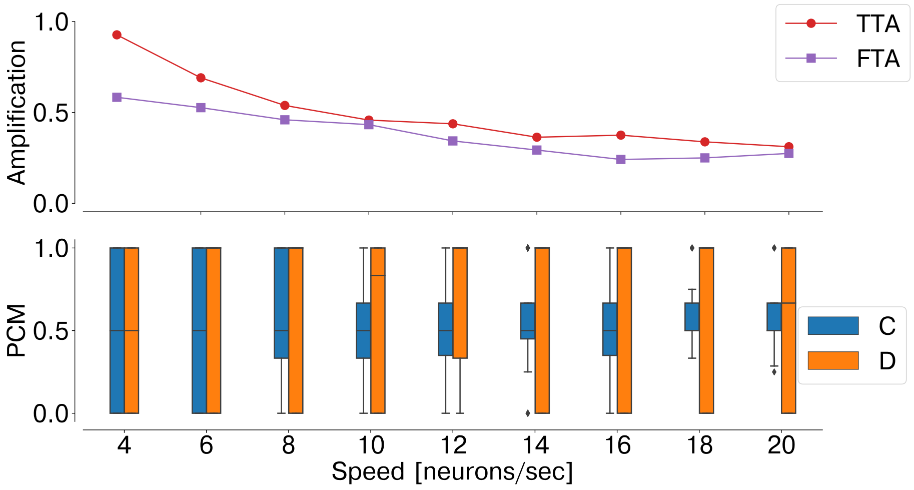

Figure S6. Performance metric sensitivity to the temporal window chosen to compute matching targets: PCM (median and interquartile range, measured across one trial over time windows t_i=10 ms), TTA and FTA. Decreasing the temporal window below the disparity neurons integration time constants (e.g. down to 10 ms) fails to capture the effective suppression of false targets in the disparity population.

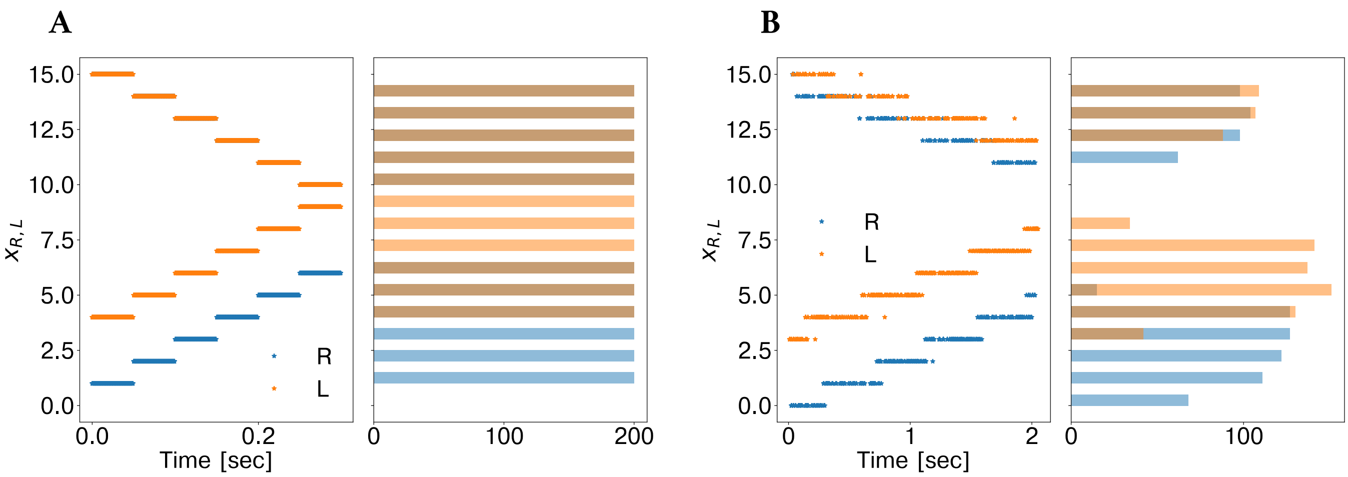

Figure S7. Input time series: synthetic input (**A**) and event camera input (**B**) projecting to network layer L2.

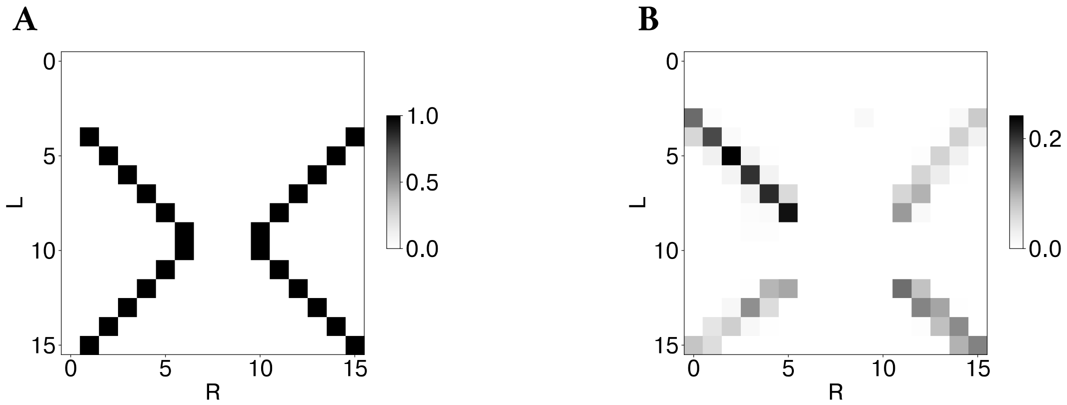

Figure S8. Spike synchrony of binocular input time series. From synthetic dataset (**A**): true matches, i.e. time series related to the same stimulus, and false matches, i.e. binocular time series related to different stimuli, yield the same degree of synchronization and therefore are not distinguishable from the temporal information only. Data from event camera and projecting to the network layer L2 (**B**): true matches yield a larger spike synchrony score.
